# Synthetic Retinoids as Potential Therapeutics in Prostate Cancer—An Update of the Last Decade of Research: A Review

**DOI:** 10.3390/ijms221910537

**Published:** 2021-09-29

**Authors:** Przemysław Hałubiec, Agnieszka Łazarczyk, Oskar Szafrański, Torsten Bohn, Joanna Dulińska-Litewka

**Affiliations:** 1 Medical Biochemistry Medical College, Jagiellonian University, 31-034 Cracow, Poland; przemyslawhalubiec@gmail.com (P.H.); agnieszka.lazarczyk@student.uj.edu.pl (A.Ł.); osk.sza2@gmail.com (O.S.); 2 Nutrition and Health Research Group 1 A-B, Department of Population Health, Luxembourg Institute of Health, 1 A-B, rue Thomas Edison, L-23 1445 Strassen, Luxembourg; torsten.bohn@lih.lu

**Keywords:** nuclear receptors, fenretinide, bexarotene, adarotene, tamibarotene, retinoid acid metabolism blocking agent, prostate cancer

## Abstract

Prostate cancer (PC) is the second most common tumor in males. The search for appropriate therapeutic options against advanced PC has been in process for several decades. Especially after cessation of the effectiveness of hormonal therapy (i.e., emergence of castration-resistant PC), PC management options have become scarce and the prognosis is poor. To overcome this stage of disease, an array of natural and synthetic substances underwent investigation. An interesting and promising class of compounds constitutes the derivatives of natural retinoids. Synthesized on the basis of the structure of retinoic acid, they present unique and remarkable properties that warrant their investigation as antitumor drugs. However, there is no up-to-date compilation that consecutively summarizes the current state of knowledge about synthetic retinoids with regard to PC. Therefore, in this review, we present the results of the experimental studies on synthetic retinoids conducted within the last decade. Our primary aim is to highlight the molecular targets of these compounds and to identify their potential promise in the treatment of PC.

## 1. Introduction

### 1.1. Overview

Prostate cancer (PC) is the second most common malignant neoplasm in men. The number of deaths caused by this disease reaches 360,000 annually [1]. Most often, it is diagnosed in subjects over 50 years of age and the progression of the disease is slow. The risk of developing PC is thought to increase significantly with age, reaching close to 100% in men >95 years. Other recognized risk factors include ethnicity and a previously diagnosed PC in close relatives (i.e., father or brother) [2].

The pathomechanisms of disease progression and the profile of molecular abnormalities in PC are complex and, to some extent, individual. The hallmark feature is the expression of erythroblast transformation specific (*ETS*) genes (most often the *ETS*-related gene (*ERG*)), which depends on androgen signaling [3]. The *ERG-TMPRSS2* translocation is highly specific for PC. However, it is not enough to explain the cancerogenesis of PC. ETS expression is accompanied by other aberrant factors, including increased stimulation of growth pathways (e.g., the frequently observed loss of phosphatase and tensin homolog activity, leading to intensified signaling in the phosphatidylinositol 3-kinase (PI3K/Akt) pathway, malfunction of deoxyribonucleic acid (DNA) repair systems (including breast cancer 1/2 (*BRCA1/2*) or checkpoint kinase-2 mutations), and loss of cell cycle control (mutated tumor protein 53) [4,5]. In addition, the frequently observed loss of functional E-cadherin (E-cad) enables cells to migrate and then metastasize.

Although the described net of molecular abnormalities is considered fundamental in regard to PC development, it must be emphasized that mutagenesis itself stems from various types of DNA damage. A strong relation exists here to reactive oxygen and nitrogen species (ROS and RNS, respectively), which can covalently modify DNA, sometimes leading to irreversible adducts and thus downstream changes via alterations in protein structure. Intracellular ROS production by nicotinamide adenine dinucleotide phosphate oxidases increases with androgen receptor (AR) activation or during an inflammatory state, both of which are observed in PC [6]. Parallel to the progression of cancerogenesis, the effectiveness of the antioxidant system decreases; thus, more mutations accumulate, and the vicious cycle continues. Examples include two antioxidant enzymes that are frequently reduced in PC due to hypermethylation of their promoters: glutathione-*S*-transferase P1 [7] and the transcription factor nuclear factor-erythroid 2 p45-related factor 2 (Nrf2), which encodes for many genes related to the bodies’ own antioxidant system [8].

However, the results of clinical studies, especially intervention trials, do not provide evidence that common antioxidant compounds (i.e., vitamin E [9] or β-carotene [10]) have any beneficial effect on either prophylaxis or treatment of PC. One proposed explanation was that these substances effectively scavenge ROS that are formed within the lipid membranes (where they reside), while they are much less effective against the hydroxyl- (HO^•^), peroxinitrite- (ONOO^−^) or superoxide anion radicals (O_2_^•−^), or hydrogen peroxide (H_2_O_2_) that dominate in prostatic tissue (cytosolic and mitochondrial ROS) [11]. Mitochondria might be the main source of ROS in PC, as they are much more abundant in PC cell lines than in a healthy prostate [12].

Nonetheless, the fundamental mechanism responsible for stimulating the growth of cancer cells in PC is the activity of AR [13]. Therefore, hormone therapy plays an important role in the treatment of advanced PC. The therapeutic options of choice include steroidal (e.g., cyproterone, abiraterone) and nonsteroidal (flutamide, enzalutamide) AR inhibitors, also surgical castration is possible. This treatment approach is relatively safe and well-tolerated by patients, however, it exhibits a significant drawback: commonly after 1–2 years of positive response to treatment, a castration-resistant PC (CRPC) develops [14]. The mechanism of this phenomenon includes mutations of the *AR*, resulting in constitutive activation (e.g., the AR-V3, V6, or V7 splice variants) and independence of androgen stimulation, increased expression of glucocorticoid receptors (which in excess may induce growth effects analogous to AR), or excessive intracellular synthesis of androgens [15].

The consequence of CRPC is to redirect the management strategy. In clinical practice, docetaxel chemotherapy is frequently introduced; however, the effectiveness remains low, and median survival reaches only about 1.5 years [16]. The search for novel treatment methods for patients at this stage of the disease has been the primary goal of PC studies in recent years.

Among the numerous substances that were investigated, promising effects were achieved in the first experiments on PC cell lines using retinoids. These compounds of natural origin are agonists of the nuclear retinoid A receptor (RAR) and retinoid X receptor (RXR). Retinoids are formed from various carotenoids as a result of oxidation by β-carotene 15, 15′-oxygenase 1; the best known and widespread representation of this reaction in the body is the transformation of β-carotene into retinal [17]. This aldehyde retinoid may then undergo further oxidation to all-trans-retinoic acid (ATRA) or can be reduced to retinol (vitamin A). The ratio of the initial carotenoid mass to the mass of retinol formed in vivo is expressed as the retinol activity equivalent index.

The scope of the importance and potential use of retinoids that can be identified in human serum under physiological conditions in PC has been thoroughly discussed in other reports [18,19]. However, recent research also covered new synthetic retinoids. These substances were synthesized on the basis of knowledge of the structure of ATRA and the mechanisms of its interaction with retinoid receptors. This review aims to summarize the last decade (from 2009 to 2021) of experimental research on these compounds with respect to PC.

### 1.2. Chemistry of Retinoids

In general, retinoids are vitamin A vitamers (the exact term of vitamin A is most often attributed to retinol itself [20]). The most commonly accepted classification of retinoids is based upon their chemical structure.

Naturally occurring substances are referred to as class I retinoids. They are composed of four isoprene units (connected in a head-to-tail manner), two of which form the trimethylated cyclohexyl ring, while the other two form a side chain terminated with a hydrophilic group (-OH, -CHO or -COOH). The molecules are highly lipophilic and of low water solubility (e.g., log P for retinol: 5.7, for ATRA: 6.3 [21]). The set of coupled double bonds enforces a planar conformation of the molecule, and the all-trans configuration is the most preferred.

The polyene double bonds provide retinoids with chromophore properties. On the other hand, the system of five double bonds increases the susceptibility of the carbon chain to oxidation and breakage, e.g., induced by ultraviolet radiation, temperature, or acids [22]. Within the organism, they are protected by proteins that carry them in serum (i.e., lipoproteins) and store them in cells (e.g., cellular retinoic acid binding protein, cellular retinoid binding protein, or fatty acid binding protein-5) [23].

Although natural retinoids are derived mainly from β-carotene, which is considered a classical antioxidant, they were thought to not possess such significant antioxidant activity. In the context of recent studies conducted by Dao et al. [24] this paradigm may not be entirely true. As an example, retinol could act as an antioxidant by donating H atoms; however, it could simultaneously produce hydroxyl radicals (HO^•^). The predominant action of retinol exposed to hydroperoxide radicals (HOO^•^) was the exergonic radical adduct formation to the C2–C3 double bond-dependent interaction between the 2p orbitals of oxygen atoms and π orbitals of the double bond (the electron transfer or H atom transfer are not thermodynamically favored). This mechanism might also be possible for other retinoids and their derivatives, although retinol was a more potent antioxidant than retinal or ATRA [24].

Class II retinoids contain one aromatic ring, while class III retinoids represent polyaromatic compounds (referred to as arotinoids). In particular, the latter class of retinoids is characterized by a selective affinity for the RAR receptor because their carbonic chain is much less flexible than that of natural retinoids (see next paragraph). This resulted from the replacement of the polyene chain with the aromatic rings placed next to the cyclohexene ring.

It is a common approach in the design of the synthetic retinoids to introduce an amide linkage to the terminal carboxylic group, which reduces the lipophilicity and facilitates the formation of hydrogen bonds. These factors are important in terms of determining the affinity to RAR receptors (particularly RARα) [25]. Such modifications characterize the structure of bexarotene, which was recently tested in PC cell lines (see the following sections). Halogen atoms (e.g., fluorine) attached to the hydrophobic benzene ring play the same role as the amide linkage. However, if a significant binding to RAR β or γ is targeted, the substitution with methyl groups in the benzene ring will be a more successful approach.

**Figure 1:**
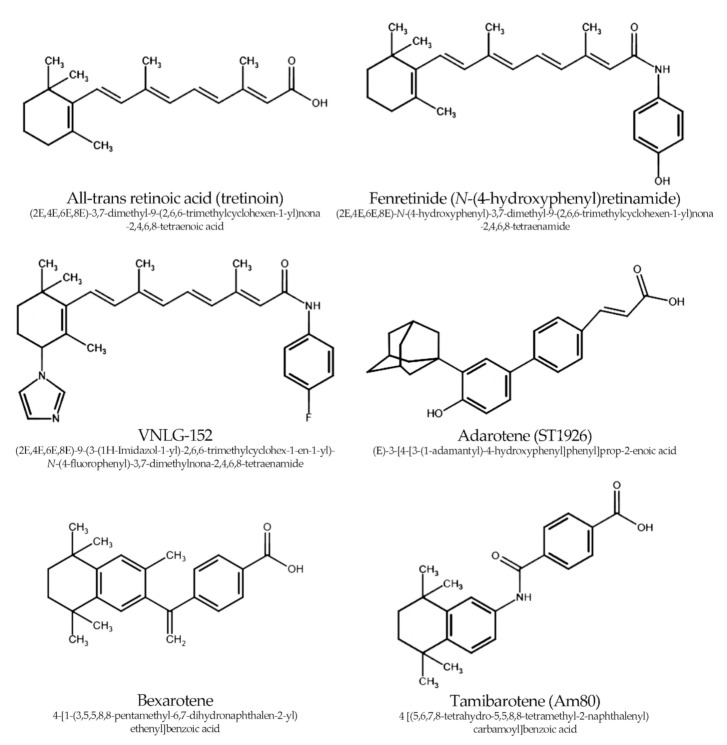
The chemical structure of ATRA and synthetic retinoids that were the main subjects of investigation in the last decade of research regarding prostate cancer.

Some studies have highlighted that low water solubility is an obstacle in developing new drugs based on the retinoid structure. Natural vitamin A is a classical lipid-soluble vitamin, and its derivatives—often containing hydrophobic benzene rings—are characterized by even higher lipid solubility. The solution to this problem is to replace the benzene ring with heteroaryl aromatic moieties [26]. The chemical structure of ATRA and the main synthetic retinoids that are discussed in this review are shown in Figure 1.

### 1.3. Retinoid Receptors

The effects of retinoids are conveyed via their interaction with the RAR and RXR nuclear receptors [27]. If RARs are devoid of their ligands, they will bind (as dimers) to retinoid response elements (RARE), initiating the recruitment of co-repressors (such as histone deacetylases or silencing mediator for retinoic acid and thyroid hormone receptor). ATRA is considered the main natural agonist of RAR; however, the receptor can also be activated by binding to synthetic retinoids [28]. 9-cis-retinoic acid (isotretinoin) was considered the physiological agonist of RXR (sometimes the term “rexinoid” is used for such molecules), yet due to the difficulties in measuring this compound in serum and most tissues, the problem is not unequivocally resolved [29]. The association of the receptor and ligand followed by binding to RARE leads to the recruitment of coactivators and induces epigenetic changes that promote transcription. The RARα, RXRα, and RXRβ receptors are highly expressed in all tissues, while the remaining retinoid receptor subtypes are rather tissue specific [30]. The RAR subtypes differ by one (α vs. β, H3 helix) or two (α vs. γ, H5 and H11 helices) amino acids. The hydrogen bond donors tend to be more selective toward the RAR subtypes α and γ, while more hydrophobic molecules have affinity to RARβ. In contrast, the ligand binding domains of the RXR subtypes do not differ in terms of their amino acid sequence.

Upon binding of the agonist, the RAR undergoes a structural change that includes movement of the H12 helix and its exposition on the surface of the receptor. This helix contains the LxxLL motif that is directly responsible for the coactivators binding and activation of histone acetylase [27]. Interestingly, if there is no ligand, then the α-helix H11 becomes the β-strand S3, which binds the corepressors. RXR forms heterodimers with other receptors (including RAR), however, ligand-bound RXR could activate the heterodimer only if the RXR partner receptor is weakly associated with its co-repressors (i.e., peroxisome proliferator-activated receptors (PPAR)), while for most of the receptors, such a situation does not occur (e.g., RAR, vitamin D receptor (VDR), or thyroid receptor (TR); these are called non-permissive heterodimers of RXR) [18].

The specificity of ligands toward RAR or RXR results from relatively simple geometric rules. The ligand-binding domain of RAR has a linear shape, while in RXR, the ligand binding pocket is L-shaped [27]. It is easy to understand how structural features of retinoids (discussed in the previous section) determine the binding specificity. The flexibility provided by the cis-conformation of the double bond in 9-cis-retinoic acid explains its affinity for both types of retinoid receptor.

Retinoids are involved in various processes taking place in the body, such as cellular differentiation (during both embryonic morphogenesis and further stages of ontogenesis), photoreceptor function of the retina, immunity, lipid metabolism, and even the metabolism of xenobiotics. Many of these are possible because RXRs are capable of forming heterodimeric pairs with the remaining nuclear receptors (e.g., AR, TR, VDR, liver X receptor, PPAR, also with RAR), thus influencing their effects. The distribution of different pairs of heterodimers varies across tissues. In the prostate gland, RXR dimerizes mainly with AR, PPARα and the estrogen receptor β. It is interesting that there exist substances specifically binding and activating only the exact heterodimer, although this was not shown for the discussed retinoids [31].

Characterizing the relationship between retinoid receptors and AR appears to be crucial in understanding the mechanisms of retinoid actions in PC. Without the ligand bound, RXR slightly increases AR signaling, but in the presence of an agonist it strongly inhibits AR actions. In turn, AR inhibits RXR expression. The androgen response element was found in the promoter region of the *RARA* gene, indicating a similar mode of regulation [32]. It should be noted that retinoids are also indirectly involved in the control of numerous stages of cholesterol synthesis, which is a substrate for steroid hormones, including testosterone.

An additional mechanism of action of the RAR receptor involves its interaction with the antioxidant factor Nrf2. Binding to ATRA increases the recruitment of the antioxidant response element (AnRE) coactivators, while unliganded RAR reduces Nrf2 activity [33]. Briefly, Nrf2 induces a large variety of mechanisms responsible for cellular self-defense against oxidative stress. The most recognized executors of Nrf2 action are heme oxygenase-1 (it removes reactive heme particles) and thioredoxin reductase 1 (it is required to recycle thioredoxin) [34], although superoxide dismutase (SOD) and catalase are downstream targets. Although in some types of cancer Nrf2 is thought to maintain cell survival and treatment resistance, in prostate tumor its levels are drastically reduced [34,35]. The result is a reduced glutathione level with elevated ROS generation and additional DNA damage.

It would be reasonable to provide concise information on the effect of RAR or RXR activation by their ligands. However, that issue is the topic of ongoing research and there is a multiplicity of RAR forms (considering that subtypes α, β and γ are further divided into isomers, thus there are nine different RAR receptors). Similarly, the effects of RXR activation strongly depend on its binding partner. The effects are usually tissue-specific and also change in diseased tissues, such as cancer. Briefly, in PC, ATRA was shown to particularly influence apoptosis, cell cycle control, and synthesis of inflammatory factors [18]. It seems that among the universal effects (i.e., observed in multiple tissues) of activation of retinoid receptors is up-regulation of SOD, which was reported in some studies [36,37]. With regard to the previous remarks on the uncertain capacity of retinoids to directly quench ROS, this might constitute another pathway via which they could stimulate antioxidant function.

The above discussed mechanisms are referred to as genomic actions of retinoids. However, they are capable of reacting directly with some proteins, which was described particularly for keratins (retinoylation). Synthetic retinoid, *N*-(4-hydroxyphenyl) retinamide (4HPR or fenretinide) was shown to increase protein retinoylation in some experimental studies [38].

## 2. Materials and Methods

Two authors have independently searched the PubMed electronic database to find relevant articles for this review.

The considered time range was from 1 January 2009 to 30 June 2021. The search formula to find the appropriate articles was: (retinoid* OR retinol OR “retinoic acid”) AND (synthetic OR derivative) AND (prostat* AND (cancer OR carcinoma OR tumor)). Original experimental studies (on cell lines or laboratory animals) that investigated the efficacy and mechanism of the action of synthetic retinoids on PC were considered for a detailed discussion. Only works published in English are covered here.

In the following, each paragraph on synthetic retinoids is started by a concise summary of the established state of knowledge based on preceding research, if available.

## 3. Synthetic Retinoids

The main findings of the studies covered by this review are summarized in Table 1.

### 3.1. Fenretinide—Experimental Studies

Multiple studies have focused on fenretinide. This retinoid analog was initially considered to have antioxidant properties that exceeded, for example, that of vitamin E (and almost threefold that of ATRA) [51]. However, the more data was acquired, the clearer the fact became that fenretinide exerts its anticancer effects instead by means of oxidative stress aggravation [52]. It was shown to trigger apoptosis in cancer cells at concentrations achievable in human serum (1.0–10.0 µM) [53]. The most established effects were: tampered mitochondrial metabolism (depolarization of the inner mitochondrial membrane) with enhanced ROS generation as well as increased ceramide production. Other targets in tumor cells, such as endoplasmic reticulum or lysosomes, were shown to be damaged by fenretinide as well. To this end, fenretinide was previously reported to lower serum insulin-like growth factor-1 (IGF-1) and reduce angiogenesis, important features in PC [52,54]. The one characteristic that supported research focused on fenretinide was that its toxicity (i.e., adverse reactions) is actually lower compared to that of natural retinoids.

Hail Jr. et al. showed that fenretinide, at 5.0 µM, induced a strong accumulation of ROS in DU145 cells, resulting in mitochondrial disruption and apoptosis. The effect was even greater (threefold) after the addition of 5.0 µM 2-heptyl-4-hydroxyquinoline-*N*-oxide (the center *i* inhibitor of complex III). On the contrary, *N*-acetylcysteine protected cells from oxidative stress caused by fenretinide. In DU145 cells, the observed changes were followed by a decrease in mitochondrial aconitase type 2, B-cell lymphoma-xL, cytochrome c oxidase subunit II and epidermal growth factor receptor, but an increase in manganese SOD. To further confirm that the effect of fenretinide depends on ROS generated during oxidative phosphorylation in mitochondria, researchers prepared DU145 cells that were devoid of mitochondrial DNA (called *ρ*^0^ clones). The administration of fenretinide to these *ρ*^0^ clones did not trigger apoptosis or significant ROS generation [39].

The crucial question to answer was at which point fenretinide triggered ROS production in the mitochondria. If it was one of the key elements of the respiratory chain (e.g., coenzyme Q or complex IV), the adverse reactions in the body after fenretinide supplementation would be more severe. However, assuming that it affected only a minor reaction of the respiratory chain (i.e., crucial only in tissues with high cellular turnover, such as cancer) the systemic toxicity would be lesser. Therefore, in another study by the same team, dihydroorotate dehydrogenase (DHODH) was investigated as a potential target of fenretinide. The enzyme is associated with the mitochondrial membrane, but it is required mainly for rapidly dividing cells, because it controls the synthesis of pyrimidines [55]. Thus, manipulation of its function creates an interesting option for the treatment of multiple types of cancers [56]. Teriflunomide (DHODH inhibitor) was shown to reduce ROS generation by fenretinide almost to the level of the control. This strongly suggests that DHODH is vital for the ability of fenretinide to generate free radicals [40]. The authors hypothesized that DHODH staining in cancer tissue could be used to predict the efficiency of fenretinide treatment. Of note, fenretinide and teriflunomide share some structural similarity, suggesting that they bind DHODH at the same site. However, teriflunomide contains a trifluoromethylphenyl moiety while fenretinide contains a phenolic one, which could be turned into a phenoxyl radical (e.g., by coenzyme Q). In fact, the role of the phenol hydroxyl group was the main interest in the next study of this team. With the model that incorporates substituted derivatives of fenretinide (natural metabolite *N*-(4-methoxyphenyl)retinamide and synthetic *N*-(4-trifluromethylphenyl)retinamide), it was determined that in fact the hydroxyl function group was essential for ROS generation [41]. This activity results in the dissipation of the mitochondrial membrane potential (ΔΨ_m_) and the release of cytochrome c.

These results corresponded well with those obtained by Benelli et al., where DU145 and PC3 cells were significantly inhibited by 10.0 µM fenretinide. Phosphorylated Fak, Akt, glycogen kinase synthase-3β (GSK-3β), cyclin D1 (CyD1), β-catenin and survivin were decreased to almost undetectable levels by fenretinide. Downregulation of Akt resulted in reduced vascular endothelial growth factor (VEGF) secretion. Along with these changes, migration and invasion were inhibited by a wide range of concentrations of fenretinide (2.5–10.0 µM) [42], suggesting a strong potency. IGF-1, which stimulates, for example, the Akt pathway and Akt phosphorylation, did not prevent these alterations. These effects were independent of ROS generation triggered by fenretinide, but stemmed from the inhibition of the Akt pathway (transfection with constitutively active, myristoylated Akt decreased the impact of fenretinide on migration). It should be noted that the regulation of the Wnt pathway (that includes β-catenin) by retinoids and specifically fenretinide depends directly on RAR/RXR signaling. This was not separately investigated by the authors, but the evidence was established years before [42,57].

Ways to enhance the activity of fenretinide were also sought. On the basis of the results from previous studies, it was established that fenretinide also acts as a stimulator of serine palmitoyltransferase and ceramide synthase. These enzymes are crucial for the synthesis of ceramide and its natural derivatives. Ceramide is processed to sphingosine-1-phosphate, which initiates carcinogenesis at higher concentrations. In this experiment, fenretinide alone significantly reduced PC3 cell viability at concentrations of 40.0 µM. Then it was administered to PC3 cells together with (2R,3Z)-*N*-(1-hydroxyoctadec-3-en-2-l)pivalamide (DM102), another synthetic agent (inhibitor of acid ceramidase). When both were administered at 10.0 µM, cell viability was reduced to about 1.5% of the control. The calculated combination index (CI) was 0.008, indicating a strong synergy between these two agents (the CI is obtained by adding the ratios of the concentrations of agents used in combination to the concentrations of agents used separately; a CI less than 1.0 indicates a synergic effect and the lower it is, the stronger the synergism). These results were followed by a 3-fold increase in caspases 3 and 7 activity (confirmed by a poly(ADP-ribose) polymerase (PARP) cleavage measurement). Ceramide levels in cells, as well as ROS generation, increased by 6-fold and 30-fold, respectively. Interestingly, the administration of vitamin E rescued cells from fenretinide-induced stress, increasing viability to 50%; whereas the ceramide synthesis was unaffected. DM102 induced cytotoxicity, which was not achieved with myriocin, the inhibitor of ceramide synthesis inhibitor. Combinations of fenretinide and DM102 were then used against DU145 cells, but the effect on cellular viability was less pronounced and the CI was higher, i.e., 0.2 [43]. This study confirmed that fenretinide alters one of the basic metabolic pathways in tumor cells. Combination with DM102 theoretically could affect cancer cells in two ways. First, fenretinide itself decreases the number of antiapoptotic proteins, lowering the apoptosis threshold level in cells (followed by the generation of ROS). Then, DM102 suppresses the effects of sphingosine-1-phospate. In addition, large amounts of ceramide cumulate and therefore generate even more ROS in a ceramide-dependent pathway. However, the experiment with myriocin showed that synergy between fenretinide and DM102 did not depend on de novo ceramide synthesis. While the impact of fenretinide on ceramide is undoubtful, the role of the ceramide pathway in PC cell survival became less clear. Thus, this study was rather the next to confirm that fenretinide leads to PC cell death primarily by induction of ROS generation. The observed synergism with DM102 also seems to depend on the aggravation of oxidative stress.

To summarize this section, we conclude that the main mechanisms of fenretinide action against PC include:

1. DHODH-dependent generation of ROS (provisionally the exclusive mechanism for generation of >90% of ROS caused by fenretinide), resulting in increased apoptosis;
2. RAR/RXR dependent down-regulation of the Wnt pathway (through a decrease in phosphorylated Akt and GSK-3β, followed by increased degradation of β-catenin), leading to reduced cell growth, migration, invasion, and neoangiogenesis.

It should be noted that the study by Gouazé-Andersson published in 2010 was the last to directly investigate the impact of fenretinide on PC cells; it was, however, used in some other studies as the reference agent. The reason was that it turned out to be almost completely ineffective in the clinical study by the Cancer Therapeutics Research Group, which also announced its results in 2010 [58] (see below). In the context of the above discussion, this might seem confusing. However, factors including low absorption after oral administration, the advancement of the disease, and selection of cell clones resistant to the treatment may have influenced the outcome of that intervention. It was even suggested that the dosage used in clinical trials was simply too low to damage the tumor cells [59]. Whether fenretinide could theoretically be used in the prevention of PC is doubtful, as the frequent adverse reactions (although mild in severity) alter its prophylactic application. Therefore, fenretinide is mainly considered a reference in the research of novel synthetic retinoids, or a structural template for them.

### 3.2. Fenretinide—Clinical Studies

Fenretinide was the only synthetic retinoid that was already considered in phase II clinical trials. Although the main goal of this review remains the discussion of experimental data, the two most recent studies on fenretinide (i.e., published in the time period covered by this paper) require a brief reflection to complete the view that we have acquired regarding the effectiveness of this molecule. A summary of these two papers is presented in Table 2.

Both studies investigated relatively small groups of elderly patients from different countries. All subjects had Eastern Cooperative Oncology Group (ECOG) performance 0–1 and usually had 0–1 comorbidity. The treatment regimens were the same and incorporated 900 mg/m^2^ of fenretinide twice daily for 1 week every 3 weeks. All the endpoints were defined according to the changes in the PSA level.

In the study conducted by Chueng et al. [60], 23 patients received a median of 5 cycles of fenretinide (range: 2–17 cycles). Only 2 patients (9%) completed the planned treatment course, while 16 (71%) of them had earlier disease progression. None of patients had PSA normalization or PSA partial response (defined as PSA decline ≥50% or ≥5 ng/mL). A total of 7 patients had PSA stable disease (with ≥90 days without PSA concentration increase), while 11 had a progression of the disease. The median time to PSA progression was 4.6 months, while the probability of not having PSA progression at 6 months of observation was 0.37 ± 0.10 (at 9 months: 0.23 ± 0.09). Furthermore, 3 patients discontinued fenretinide due to serious adverse reactions. In one patient, the reason was preexisting renal dysfunction (elevated creatinine); however, in the remaining patients, newly diagnosed thrombocytopenia and nyctalopia occurred. Other grade 3 toxicities were fatigue, hypermagnesemia, and increased intestinal lipase activity. The main conclusion of this investigation was that fenretinide had little efficacy against PC.

The main difference in the design of the Cancer Therapeutics Research Group [58] trial was that its 27 patients had advanced, metastatic (80% had bone metastases, 31% had soft-tissue metastases) PC. The patients received a median of 2 cycles (range: 0–11). Tumor progression occurred in 22 patients, while 3 subjects terminated treatment due to its toxicities (including a grade 3 toxicity, a rash). The median time to failure of fenretinide treatment was 54 days. Only one PSA response (lasting 39 days) was observed. Authors strictly concluded that continuation of research on fenretinide in refractory prostate cancer is not encouraged.

As mentioned above, these results actually led to the termination of all investigations on fenretinide with respect to PC management. In fact, today one may find them less convincing for several reasons. First, both studies based their conclusions solely on the level of PSA, that is, “biochemical recurrence”. However, it was established about a decade ago that the biochemical recurrence exhibits no clear association with clinical results (e.g., overall survival) and has low diagnostic value (low sensitivity and specificity) [61,62]. In fact, fenretinide-induced oxidative stress could result in increased PSA without any progression of PC [63]. Considering the small groups of patients, an important bias might have been introduced here.

The second drawback that must be pointed out is that both studies lacked any control group and randomization. Therefore, there was no control over the random confounding factors that might have potentially influenced the results [64]. A randomized controlled trial involving a second group of patients who received docetaxel (a control group with standard treatment for CRPC) would provide more credible evidence.

Therefore, it is not entirely clear whether the withdrawal of fenretinide from PC research was justified. In fact, the unfavorable results of clinical trials investigating other cancers suggest that fenretinide is not effective in the clinical setting [65]. However, in the last few years new trials on fenretinide did start. They implemented novel drug delivery systems and treatment regimens. None of the currently running studies investigates PC. However, regarding the above considerations, it would be interesting to see if novel randomized controlled trials confirm the results obtained in the previous decade.

### 3.3. RAMBAs

A group of novel C-4 heteroaryl 13-cis-retinamides (derivatives of 13-cis-retinoic acid) was synthesized and investigated by Mbatia et al. [44]. Another name for those novel retinamides is “retinoid acid metabolism blocking agents” (RAMBA), as they inhibit a group of cytochrome P450 enzymes, which process ATRA. Against PC cells, derivatives with an imidazole group in the C-4 position and different terminal amide components (e.g., -H, -OH, -F in different positions of the benzene ring) were employed. The concentration range used was 0.6–20.0 µM for 24 h. Two of the most promising lead compounds (for which most effects were observed at 5.0 µM) were the VNMH-1-73 and VNMH-1-81 retinoids, substituted with p-OH and m-F in the benzene ring of terminal aryl amide, respectively. The postulated mechanism of their action included the inhibition of mitogen-activated protein kinase interacting kinases 1 and 2 (Mnk1/2; with reduced eukaryotic translation initiation factor 4E (eIF4E) phosphorylation) and degradation of AR and its V7 splice variant. AR activity upon dihydrotestosterone stimulation was also reduced. In addition, B-cell lymphoma-2 (Bcl-2) and CyD1 were depleted, while the concentration of cleaved PARP (a marker of apoptosis) and Bcl-2-associated agonist of cell death were elevated. For each derivative, the half-maximal inhibitory concentration (IC_50_) values were calculated and compared to ATRA or fenretinide. Against LNCaP cells, the most effective was VNMH-1-81, showing IC_50_ values of 1.69 µM (ATRA – 47.9 µM; fenretinide – 2.7 µM). The same compound presented the lowest IC_50_ for PC3 cells, namely IC_50_ values of 3.5 µM (ATRA – 36.3 µM; fenretinide – 3.5 µM). Among all synthetic retinoids tested, seven were more effective in reducing PC cell growth than ATRA. However, fenretinide was shown to exert a similar influence to that of the most prominent of the derivatives. Generally, PC3 cells seemed to be the most resistant to C-4 heteroaryl 13-cis retinamides. 1*H*-imidazole as the heteroaryl ring was associated with the highest anticancer activity. Furthermore, VNHM-1-73 at 20 mg/kg for 1 month reduced tumor graft growth of the CWR22Rv1 graft in the mice model (to 38.8% of the control). Based on additional findings, it was suggested that translation rather than the cell cycle itself is the primary target for these compounds.

Ramamurthy et al. continued to develop RAMBA in their study. The newly introduced agents—VNLG-145, VNLG-147, VNLG-152, and VNLG-153—were much more effective against both LNCaP (with the lowest IC_50_ being 3.0 µM for VNLG-147, which is an o-OH substituted derivative similar to that investigated by Mbatia et al.) and PC3 (with the lowest IC_50_ being 1.9 µM for VNLG-147). VNLG-153 remained the most effective in reducing 22Rv1 growth. Also, CyD1 was reduced, similar to cyclin B (CyB), resulting in cell cycle arrest (74.6% of G_1_ cells for VNLG-152 compared to 37.1% in the control). Additional experiments with a caspase inhibitor (ZVAD) showed that RAMBA-triggered apoptosis was completely dependent on caspases activity. Furthermore, after treatment with VNLG-152, migration and invasion were reduced, which was attributed to decreased N-cadherin (N-cad) and restoration of E-cad (VNLG-147 and 153 also had such an effect, although weaker). Additional experiments showed that RAMBAs act mainly through promoting proteasomal degradation of fAR and Mnk1, which is mediated by the E3 ubiquitin ligases mouse double minute 2 homolog and C-terminus of Hsc70-interacting protein [45]. The authors decided that VNLG-152, due to its outstanding properties, would be their lead compound and focused on it in their more recent work.

In their latest study, 22Rv1 cells were injected into mice as an animal model of PC [46]. The rodents were then given 10 or 20 mg/kg/day of VNLG-152 for 5 days per week. The tumor size was reduced to 63.4% and 76.3% of the control, respectively. Importantly, no systemic or organ toxicities were observed after VNLG-152 administration. The results of the measurements in grown tumor tissues were concomitant with those of the in vitro studies. The higher dose of VNLG-152 caused >90% reduction in AR-V7, Mnk1/2, and p-eIF4E levels. The influence exerted on the Mnk-eEIF4E system requires particular attention. It was earlier shown, that eIF4E phosphorylated on Ser 209 augmented the resistance to oxidative stress in tumor cells [66]. Therefore, substances that eliminate its presence effectively reduce the proliferation of tumor cells. This seems to be particularly true, as simultaneous inhibition of Mnk1/2 leads to a switch to oxidative metabolism [67], which is associated with ROS production in cancer cells.

The in vitro investigation of 22Rv1 cells showed additionally that the other phosphorylated proteins important for translation processes were decreased (namely phosphorylated mechanistic target of rapamycin (p-mTOR) and phosphorylated 4E-binding protein (p-4E-BP1)). On the contrary, 4E-BP1, which antagonizes the eIF4G-Mnk subcomplex, was increased. Only for fAR was the decrease less pronounced and reached about 40%. The stimulation with 10.0 nM dihydrotestosterone was not able to counterbalance the effects of VNLG-152 on the AR activity [46]. A remarkable effect induced by VNLG-152 was the suppression of the epithelial-mesenchymal transition, associated with the repression of N-cad, β-catenin, claudin, slug, snail, twist, vimentin, matrix metalloproteases 2 and 9 (MMPs), as well as an increase in E-cad.

Almost a decade of research on RAMBAs resulted in the promotion of VNLG-152 as the most potent agent. Today it is widely tested, not only against CRPC, but also in triple-negative breast cancer and other tumors, which are currently thought to be incurable [68]. It presents at least three outstanding properties:

1. It induces the degradation of AR (and preferentially its AR-V7 splice variant, responsible for resistance to treatment with enzalutamide and abiraterone);
2. It impairs the process of protein synthesis by promoting Mnk1 degradation and then blocks the phosphorylation of eIF4E (and possibly other proteins important for translation, for example, mTOR);
3. It reverses the molecular changes responsible for the epithelial-mesenchymal transition, showing the capacity to reduce tumor invasion and metastases in vivo.

Additionally, knowing the structure of VNLG-152, one may try to implement other modifications, e.g., replacing fluorine with other halogens or adding more fluorine atoms to the benzene ring. The future will show whether VNLG-152 or its derivatives join the list of approved chemotherapeutics for PC treatment. Its combination with other anticancer drugs might turn out to be crucial for PC therapy.

### 3.4. Bexarotene

The one synthetic retinoid, which has already found an application in chemotherapy (mainly of cutaneous T-cell lymphoma) is bexarotene, a class III retinoid and selective ligand of RXR. Previously, it was shown to be effective in combination with chemotherapeutics in other cancers (e.g., non-small-cell lung carcinoma). Moreover, it reduced the rate of developing resistance to chemotherapy in PC3 cells [69]. However, since then, it was not specifically investigated in PC research until 2019. It stemmed from the theoretical concern that bexarotene, due to the activation of RXRα, will inhibit Nrf2 (which is already down-regulated in PC). Such a dose-dependent relationship between bexarotene and Nrf2 was truly identified [70]. However, the other pathways that control the antioxidant defense system must be considered. In fact, the interaction of bexarotene-bound RXR with PPARγ results in the recruitment of sirtuins (SIRT; like SIRT1 or SIRT6) [71,72]. These nicotinamide adenine dinucleotide-dependent histone deacetylases play a well-established role in the protection from ROS and modulation of genes responding against oxidative-stress [73]. Such considerations resulted in the reappraisal of bexarotene in PC.

The idea of Shen et al. was to investigate whether bexarotene could trigger a synergistic effect together with docetaxel and thus increase the effectivity of first-line CRPC treatment. In fact, a combination of 10.0 nM docetaxel and 40.0 µM bexarotene resulted in a 60% decrease in the proliferation in PC3 cells and a 90% reduction in it in DU145 after exposure for 24–48 h. IC_50_ for bexarotene (alone) was calculated as 40.6 ± 0.45 μM for PC3 and 50.2 ± 4.1 μM for DU145. Bexarotene proved to reduce levels of CyD1 and E2 (with no effect on CyB1, cyclin-dependent kinase 1 and phosphorylated histone 3), and despite its failure to induce the G_2_/M cell cycle arrest, acted synergistically with docetaxel in that respect (in both cell lines) [47]. Interactions of bexarotene and docetaxel were also investigated by Hu et al. [48], in order to find whether targeting of testicular nuclear receptor 4 (TR4) explained the mechanism of the above-mentioned synergism. What is interesting is that retinoids might directly fill the TR4 ligand binding pocket to antagonize it (as it was shown by analysis of the crystalline structure of the receptor for ATRA) [74]. The authors excluded the idea that the action of bexarotene depended only on the activity of RXR because it was effective only at concentrations greater than 8.0 µM (while it should already activate RXR, at least assuming that it will act on the permissive receptor heterodimer). Furthermore, bexarotene effectively reduced docetaxel-resistant cell proliferation (these cells must have expressed high levels of TR4) but did not significantly affect parental cells.

The investigation of multiple PC cell lines (PC3, DU145, C4-2, and 22Rv1) and PC specimens from clinical tissues was performed thereafter. It was revealed that blocking TR4 actions with 6.0 µM bexarotene (for 24 h) resulted in the mitigation of lincRNA-p21/hypoxia-inducible factor-1α (HIF-1α)/VEGF signaling, causing an enhanced susceptibility to docetaxel [48]. The mitigation of HIF-1α signaling is a crucial element of anticancer strategy, as this factor is responsible for the resistance to damage caused by oxidative stress [75]. In fact, the relationship between HIF-1α and the previously mentioned Nrf2-AnRE pathway might be also important here. Regarding the HIF-1α promoter, it was shown that the HIF-1α promoter has an AnRE element, which enhances the transcription of this factor [76]. Although the authors exclusively discussed TR4 effects, the mechanism involving RXRα actions was not mechanistically excluded. In that context, it is possible that inhibition of Nrf2 (through RXRα), observed earlier in bexarotene studies, could become important for the drug synergy between bexarotene and docetaxel, although investigated alone would be rather unfavorable (i.e., Nrf2 deficiency alone is associated with the progression of PC).

In brief, the knowledge of bexarotene’s role in PC is currently restricted to its interaction with docetaxel. The main bexarotene-dependent mechanisms for increased susceptibility of cancer cells to this taxane are:

1. inhibition of the expression of CyD1 and E2 expression;
2. TR4 antagonism followed by inhibition of the lincRNA-p21/HIF-1α/VEGF downstream pathway.

The role of RXR receptor signaling (including the blocking of the Nrf2 pathway) in bexarotene actions against PC cells was questioned; however, no strict conclusions can be drawn at this moment.

### 3.5. Other Synthetic Retinoids

A novel approach introduced in 2019 was the application of the atypical adamantyl retinoid adarotene (ST1926). In previous studies, it was reported to possess strong anticancer activity and trigger fewer adverse events than natural retinoids [77].

It reduced the proliferation of ATRA resistant DU145 cells (both used at concentrations of 1.0 µM and 10.0 µM concentrations) and caused the accumulation of PC3 and DU145 cells in the sub-G_1_ and S phase of the cell cycle, with massive DNA fragmentation in PC3 cells. The pathway involving p53 showed to be crucial for its action. Additionally, phosphorylated histone H2AX (γH2AX; a marker of double-strand DNA breakage [78]) was elevated after adarotene treatment. Finally, adarotene targeted and eliminated cancer stem cells in a time- and dose-dependent manner in sub-µM concentrations (0.01 µM) in the sphere formation assay [49]. Additionally, it reduced the migration and invasion of cancer cells at 1.0 µM for 48 h. At the same dosage, it increased caspase activity, which was reflected in elevated cPARP. Of note, tumor growth and progression were reduced also in vivo in a mouse model. Sections taken from the tumors were shown in fluorescence microscopy to have reduced Ki-67 expression, followed by a decrease in lineage epithelial markers (cytokeratin 8 and 14) and stemness markers (cluster of differentiation 49f and 44).

Notwithstanding the question to which extent these effects would be maintained in the environment of the human body (or do not trigger adverse reactions due to the presence of adamantane fragments), this preliminary work suggests to further assess its application in chemotherapy of PC.

The most recent study that is discussed here was conducted by Ishigami-Yuasa et al. in 2019 [50]. The authors investigated tamibarotene (Am80), a specific RARα/β agonist, together with a histone deacetylase (HDAC) inhibitor (suberoylanilide hydroxamic acid (SAHA)) or DNA methyltransferase inhibitor 5-aza-2′-deoxycytidine (5-AzadCyD). Tamibarotene, known also as retinobenzoic acid, was initially synthesized to support the treatment of acute promyelocytic leukemia resistant to ATRA [79].

The idea behind the study design was that blockage of HDAC and DNA methyltransferase will lead to the restoration of RAR expression, thus potentiating the impact of the retinoids. The cell lines used were LNCaP and PC3. The IC_50_ values for tamibarotene were relatively high (36.0 µM and 52.0 µM respectively). A kind of synergy against LNCaP was shown when tamibarotene and SAHA (or 5-AzadCyD) were administered at high concentrations, with reduction in prostate-specific antigen collected from cell supernatant. However, the same synergy was only noticeable in terms of apoptosis (38.1% compared to 35.5% for tamibarotene alone). Additional research with KD5170, a specific class IIB HDAC inhibitor (HDAC6 and 10) suggested that class IIB is of major importance for interaction with tamibarotene. Finally, each of the investigated agents (tamibarotene, SAHA and KD5170) increased the level of RARα. The paradox was that the combination of tamibarotene with either of these two caused a significant depletion of RARα. The only hypothesis to explain this phenomenon was that the receptor, when bound to a ligand, triggers a negative-feedback loop (such as the deacetylation of heat-shock protein 90 resulting in degradation of RARα), which is strengthened by HDAC inhibitors.

Tamibarotene will undergo further investigations that will resolve whether these ideas are correct. At this moment, using the simple measure of IC_50_, it seems inferior to fenretinide, RAMBAs, or adarotene. Furthermore, HDAC inhibitors are not considered the best choice for the management of CRPC, and their use in PC was already questioned after the publication of results of several clinical trials of phase II and III [80].

## 4. Conclusions

The last decade of research has brought about a number of novel derivatives of natural retinoids, some of which presented properties superior to those of their maternal molecules. The most promising of them are RAMBA VNLG-152 and adamantyl retinoid adarotene. The first targeted multiple entities of critical importance in PC pathobiology, such as AR (with its V7 variant), the Mnk1/2 pathway or the epithelial-mesenchymal transition, while the latter appeared to target the cancer stem cell population.

The role of bexarotene and its remarkable synergism with docetaxel requires attention as well. There is a lot of clinical experience with this synthetic retinoid, thus it could be introduced into practice of PC management in a rapid way, provided that the initial results presented here will be followed by appropriately-designed, prospective clinical trials.

Researchers should be encouraged to apply a search strategy similar to that of Ramamurthy et al. during their investigation for the most efficient lead compound [45], i.e., the gradual selection of the most potent compound from a wide series of potential substances. Such an approach increases the probability that the derivative of the highest potency against PC will be ultimately identified.

In addition to this, future clinical trials must be carefully designed with attention being paid to specific aspects of PC biology (e.g., dependence between PSA and the clinical course of the disease). Primary clinical outcomes such as survival or symptoms should be considered first, to provide conclusive results that will not be questionable. A properly designed randomized controlled trial could, for example, compare the time to clinical progression or survival in subjects treated with docetaxel alone or with bexarotene. Similar studies could be conducted with VNLG-152 or adarotene after initial safety assessments.

## Figures and Tables

**Table 1:** Overview of results of laboratory studies investigating the impact of synthetic retinoids on prostate cancer (cell lines, PC tissue or mice model).

Compound Name	Experimental Model	Concentration Range or Dose Used	Investigated Feature	Results	Comment	Reference
fenretinide (4HPR)	(1) DU145 (2) DU145 lacking mtDNA (*ρ*^0^ clones)	5.0 µM for 24 h (for some experiments the time of culturing with fenretinide was 1–2 h or up to 48 h)	Apoptosis ROS generation Immunoblot	(1) 80% hypoploid cells vs. 20% in control ACO2↓50%, Bcl-xL↓40%, COII↓50%, EGFR↓55%, MnSOD↑40% (β-actin n/c) ROS generation rate was 6.5-fold higher than in control (2) hypoploidy was similar to control (10–15%) ROS generation rate was similar to the control	The main results of the study suggest that the anti-cancer effects of 4HPR in PC depend strongly on the ROS generation during oxidative phosphorylation. ROS generation was 3-fold greater after addition of 5.0 µM HQNO (compared to 4HPR alone). The generation of ROS and mitochondrial disruption was reduced by NAC.	Hail Jr. et al., 2009 [39]
fenretinide (4HPR)	(1) LNCaP (2) PC3 (3) DU145	5.0 µM for 150 min	ROS generation rate Apoptosis	4HPR induced ROS production in DU145 6-fold greater in compared to the control inhibition of DHODH by TFN caused reduction of 90% ROS generation, to similar level to the control	Probably the main mechanism of 4HPR is generation of ROS mediated by DHODH.	Hail Jr. et al., 2010 [40]
fenretinide (4HPR), fenretinide metabolite 4MPR, fenretinide derivative 4TPR	LNCaP	10.0 µM for 24 h (the time of culturing for DiOC_6_ retention assessment was 6 h)	Apoptosis ROS generation	60% preapoptotic cells after 4HPR; to 5% of apoptotic cells in control and for 4MPR or 4TPR 80% hypoploid cells vs. 20% in control 4HPR induced ROS generation rate was 9.0× higher the in control; 4MRP and 4TPR were similar to control	The study design incorporated two (natural and synthetic) analogs of 4HPR that were devoid of free hydroxyl function group. As these substances were ineffective in induction of both ROS generation and apoptosis, it seems likely that hydroxyl group is vital for pharmacological activity of 4HPR.	Hail Jr. et al., 2010 [41]
fenretinide (4HPR)	(1) DU145 (2) PC3	2.5–10.0 µM for 4–96 h	Cell growth Migration Invasion Immunoblot	(1) cell growth after 24 h similar to control for all concentrations; after 96 h reduced to about 50% of control for 10.0 µM 4HPR migration after 96 h of exposure reduced by 2.5/5.0/10.0 µM 4HPR to 70/70/50%, respectively invasion after 96 h of exposure reduced by 2.5/5.0/10.0 µM 4HPR to 80/50/50%, respectively p-Fak↓, p-Akt↓, β-catenin↓, p-β-catenin↓, p-GSK-3β↓, CyD1↓, survivin↓after 4 h VEGF secretion↓after 16 h (2) cell growth after 24 h similar to control for all concentrations; after 96 h reduced to about 40% of control for 10.0 µM 4HPR migration after 96 h of exposure reduced by 2.5/5.0/10.0 µM 4HPR to 80/50/50%, respectively (effects independent of ROS production) invasion after 96 h of exposure reduced by 2.5/5.0/10.0 µM 4HPR to 60/40/30%, respectively p-Fak↓, p-Akt↓after 4 h VEGF secretion↓after 16 h	ROS scavengers such as 10.0 mM NAC or 1.0 µM DPI did not reduce the migration inhibiting effect of 4HPR in PC3 cells although 4HPR-dependent ROS production is significantly reduced. NAC do not change 4HPR’s ability to modulate β-catenin signaling. The specific PI3K/Akt inhibitors (200.0 nM wortmannin and 10.0 µM LY294002) act synergistically with 4HPR in reduction of cell migration. In DU145 4HPR reduces effect of stimulation with 100 ng/mL IGF-1 on migration and p-Akt level. Cells transfected with constitutionally active Myr.Akt are not susceptible to these effects.	Benelli et al., 2010 [42]
fenretinide (4HPR)	(1) PC3 (2) DU145	0.0–50.0 µM for 72 h (in the most of experiments 4HPR was used together with DM102)	Cell viability Combination index CASPs activity cPARP Ceramide level ROS generation	(1) cell viability was reduced with concentrations higher than 30.0 µM of 4HPR CIs for 2.5/5.0/10.0 µM 4HPR and 10 µM DM102 were 0.061/0.021/0.008, respectively CASP3/7 activity n/c; if 4HPR was given together with DM102 CASP3/7 activity↑200–300% cPARP↑ ceramide↑6× after 24 h exposure to 10.0 µM 4HPR ROS level↑30× after 24 h exposure to 10.0 µM 4HPR (2) 25.0 µM 4HPR reduced cell viability to 78%; combined with 15.0 µM reduced cell viability to 15% (CI 0.2)	4HPR and DM102 synergistically reduce the viability of PC3 cells. Synergy of 4HPR and NOE was observed only for high concentrations of NOE (50 µM). CASPs activity, ceramide level, and ROS production are higher after simultaneous exposure to 4HPR and DM102. Myriocin failed to rescue PC3 cells from the cytotoxicity induced by the combination of these compounds. 250.0 µM of vitamin E reduced the cytotoxic effect of 4HPR on PC3 cells.	Gouaze’-Andersson et al., 2011 [43]
lead compounds: (A) VNHM-1-81 (B) VNHM-1-73^1^	(1) LNCaP (2) CWR22Rv1 (3) PC3 (4) castrated mice bearing CWR22Rv1 xenografts	0.6–20.0 µM for 24 h	Cell growth Immunoblot Migration	(A) IC_50_ (µM) in LNCaP/CWR22Rv1/PC3: 2.69 ± 0.14/2.04 ± 0.01/5.62 ± 0.03 in LNCaP fAR↓(to 0% at 20.0 µM), Mnk1↓, Mnk2 n/c, p-eIF4E↓, CyD1↓, cPARP↑(β-actin n/c) cells migration reduced DHT-dependent AR signaling was 7-fold reduced in LNCaP after 18h treatment with 10.0 µM VNHM-1–81 (B) IC_50_ (µM) in LNCaP/CWR22Rv1/PC3: 1.69 ± 0.07/1.86 ± 0.06/3.54 ± 0.02 in LNCaP fAR↓(to 0% at 15 µM), Mnk1↓ (to 0% at 10.0 µM), Mnk2↓, p-eIF4E↓(to 0% at 5.0 µM), CyD1↓, cPARP↑(from 10.0 µM) (β-actin n/c) cells migration reduced DHT-dependent AR signaling was 2-fold reduced in LNCaP after 18h treatment with 10.0 µM VNHM-1–73 in mice model treatment with 20 mg/kg of VNHM-1-73 for 5 days per week for one month resulted in %T/C equal to 38.8% (*p* = 0.0001); immunoblot of cancer tissue showed: AR↓, AR-V7↓, Mnk1/2↓, eIF4E n/c, p-eIF4E↓, CyD1↓, Bcl-2↓, Bad↑(GAPDH n/c)	LNCaP cells transfected with si-AR and/or si-Mnk1 did not show an effect of treatment with VNHM-1-81. Supposing that VNHM-1-81 acts on them at the post-transcriptional stage.	Mbatia et al., 2015 [44]
lead compound VNLG-152^2^	(1) LNCaP (2) CWR22Rv1 (3) C4-2B	0.6–20.0 µM for 24 h	Apoptosis Cell growth Colony formation Immunoblot Cell cycle	after exposure to 5.0 µM VNLG-152 apoptosis in LNCaP cells was 2.5× of observed in control colony formation reduced to 15–25% of control (in LNCaP, CWR22Rv1, C4-2B) by 5.0 µM VNLG-152 after exposure to 10.0 µM VNLG-152 (results compared to control for LNCaP/CWR22Rv1/C4-2B, respectively): Mnk1↓(7/7/9%), AR↓(12/22/51%), eIF4E↓(5/9/8%), p-eIF4E^ser209^↓(9/5/8%), PSA↓(24/11/17%) (β-actin n/c) after exposure to 5.0 µM VNLG-152 (results compared to control for LNCaP/CWR22Rv1/C4-2B, respectively): CyD1↓(18/53/10%), CyB↓(3/53/18%), Bax↑(350/390/350%), cPARP↑(450/660/510%)	AR and Mnk1 are the most important targets of VNLG-152. They are reduced through the posttranslational mechanism.	Ramamurthy et al., 2015 [45]
VNLG-152	CRPC tumor xenograft model (CWR22Rv1 cells in castrated mice)	10 or 20 mg/kg, twice daily, for 5 days	TGI Immunoblot	VNLG-152 vs. vehicle: TGI 63.4% (at dose 10 mg/kg) or 76.3% (at dose 20 mg/kg) at dose 10 mg/kg: AR-V7↓70%, Mnk1/2↓90%, p-eIF4E↓60%, PSA↓60%, CyD1↓50%, Bcl-2↓75%, Bax↑500%, CASP3↑1500%, cPARP↑500%, E-cad↑250%, N-cad↓10%, β-catenin↓50%, claudin n/c, Snail↓5%, Slug↓20%, Twist↓50%, vimentin n/c, MMP2/9↓90–95% at dose 20 mg/kg: AR-V7↓95%, Mnk1/2↓90%, p-eIF4E↓80%, PSA↓90%, CyD1↓55%, Bcl-2↓90%, Bax↑1250%, CASP3↑1500%, cPARP↑500%, E-cad↑300%, N-cad↓80%, β-catenin↓99%, claudin↓90%, Snail↓50%, Slug↓50%, Twist↓80%, vimentin↓10%, MMP2/9↓90–95%	VNLG-152 blocks the pathways responsible for EMT.	Ramamurthy et al., 2018 [46]
bexarotene	(1) PC3 (2) DU145	20.0–40.0 µM for 24–48 h (combined with 5.0–10.0 nM docetaxel)	Cell cycle Apoptosis Immunoblot	bexarotene caused cell cycle arrest in G_1_ phase at 40.0 µM in both cell lines IC_50_: in PC3 40.6 ± 0.5 μM, in DU145 50.2 ± 4.1 μM CyB1↓, CDK1↓ in both cell lines (1) percentages of apoptotic cells: DMSO (control): 1.66%, bexarotene at 20.0 µM: 3.28%, bexarotene at 40.0 µM: 3.11%, bexarotene at 20.0 µM + docetaxel at 10.0 µM: 3.3%, bexarotene at 40.0 µM + docetaxel at 10.0 µM: 4.14% (2) percentages of apoptotic cells: DMSO (control): 4.67%, bexarotene at 20.0 µM: 9.9%%, bexarotene at 40.0 µM: 10.2%, bexarotene at 20.0 µM + docetaxel at 5.0 µM: 18.2%, bexarotene at 40.0 µM + docetaxel at 5.0 µM: 17.71%	Bexarotene acts synergistically with docetaxel in lines representing CRPC. The mechanism involves inhibition of CyB1 and CDK1.	Shen et al., 2019 [47]
bexarotene	(1) PC3 (2) DU145 (3) C4-2B (4) CWR22Rv1 (5) clinical samples of PC tissues	0.0–24.0 µM for 24 h (combined with 0.0–400.0 nM docetaxel)	TR4 antagonism	TR4↑ after docetaxel chemotherapy chemoresistance was reduced after suppressing TR4 with bexarotene bexarotene at doses <8.0 μM did not influence cell proliferation in PC3 and DU145 bexarotene at 8.0 μM increased chemosensitivity of PC3 and DU145 cells with overexpression of TR4 and decreased proliferation of chemoresistant CWR22Rv1 and C4-2B cells	TR4 might be elevated in docetaxel-resistant PC. Targeting TR4/lincRNA-p21/ HIF-1α/VEGF signaling with bexarotene may increase the prostate cancer cells’ chemo-sensitivity to docetaxel.	Hu et al., 2020 [48]
adarotene (ST1926)	(1) DU145 (2) PC3 mouse PC cell lines: (3) PLum-AD (4) PLum-AI (5) PC xenografts in mice	0.5–10.0 µM for 48 h	Cell growth Invasion Migration Cell cycle Apoptosis Sphere formation	cell growth reduced (in DU145/PC3/Plum-AD/Plum-AI, respectively) by 1.0 µM ST1926 to: 60/65/10/30% and by 10.0 µM ST1926 to: 50/50/5/25% number of migrating cells reduced by 1.0 µM ST1926 to 1000 (vs. 270 in control) in DU145 and to 500 (vs. 2500) in PC3 the percentage of sub-G_1_ increased by 1.0 µM ST1926 to 40% (vs. 10% in control) in DU145 and to 30% (vs. 10% in control) in PC3; arrest in S phase induced in 30% (vs. 15% in control) of DU145 and in 25% (vs. 15% in control) of PC3 the percentage of sphere forming colonies after 11 days of treatment with 0.01 µM ST1926 (1st sphere generation): 9% (vs. 12% in control) in DU145–mean sphere diameter 45 µm, 8% (vs. 12% in control) in PC3–mean sphere diameter 80 µm	ST1926 attenuates ATRA-resistant prostate cancer cells’ growth and potentially targets prostate cancer stem-like cells.	Bahmad et al., 2019 [49]
tamibarotene (Am80)	(1) LNCaP (2) PC3	(1) 0.1–100.0 µM for 72 h to IC_50_ assessment (2) 25.0 µM for synergy studies (with 1.6–2.6 µM SAHA and 9.5–13.0 µM 5-AzadCyD) for 24–72h	Growth inhibition Apoptosis Immunoblot PSA in cell culture supernatant HDAC activity	IC_50_ in LNCaP: 36.0 µM, in PC3: 52.0 µM synergy with SAHA or 5-AzadCyD against LNCaP (with ↓PSA), but not against PC3 Am80 + SAHA increased apoptosis from 35.5% (Am80 alone) to 38.1% RARα↑70% (for Am80 + SAHA:↓20%)	The synergy between tamibarotene and SAHA results from inhibition of class IIB HDAC. Tamibarotene alone or SAHA alone increase the expression of RARα, however, combined they decrease it.	Ishigami-Yuasa et al. 2019 [50]

Note: 1VNHM-1–81 and VNHM-1–73 showed the most outstanding properties of 18 new C-4 heteroaryl 13-cis-retinamide Mnk/AR degrading agents tested by Mbatia et al. Data for the other compounds are not discussed in detail and are not shown here. 2VNLG-152 showed the most outstanding properties of 8 new RAMBAs tested by Ramamurthy et al. Data for the other compounds are not discussed in detail and are not shown here. Abbreviations: %T/C—median tumor volume of treated group to median tumor volume of control group, 4HPR—N-(4-hydroxyphenyl) retinamide or fenretinide, 4MPR—N-(4-methoxyphenyl)retinamide, 4TPR—N-(4-trifluromethylphenyl)retinamide, 5-AzaCyD—5-aza-2′-deoxycytidine, ACO2—aconitase type 2, AR—androgen receptor, ATRA—all-trans-retinoic acid, Am80—tamibarotene, Bcl-XL—B-cell lymphoma-extra large, Bad—Bcl-2-associated agonist of cell death, Bax—Bcl-2-like protein 4, Bcl-2—B-cell lymphoma 2, CDK1—cyclin-dependent kinase 1, CASP—caspase, CI—combination index, COII—cytochrome c oxidase subunit II, CRPC—castration resistant prostate cancer, CyB—cyclin B, CyD1—cyclin D1, DHODH— dihydroorotate dehydrogenase, DHT—dihydrotestosterone, DMSO—dimethyl sulfoxide, DPI—diphenyleneiodionium chloride, DiOC6—3,3′-dihexyloxacarbocyanine iodide, E-cad—E-cadherin, EGFR—epidermal growth factor receptor, EMT—epithelial-mesenchymal transition, Fak—focal adhesion kinase, GAPDH—glyceraldehyde 3-phosphate dehydrogenase, GSK-3β—glycogen kinase synthase-3β, HDAC—histone deacetylase, HIF-1α—hypoxia-inducible factor-1α, HQNO—2-heptyl-4-hydroxyquinoline-N-oxide, IC50—half maximal inhibitory concentration, IGF-1—insulin-like growth factor-1, MMP—matrix metalloprotease, MnSOD—manganese superoxide dismutase, Mnk1/2—mitogen-activated protein kinase interacting kinases 1 and 2, Myr.Akt—myristoylated Akt, NAC—N-acetyl cysteine, N-cad—N-cadherin, NOE—N-oleoylethanolamine, PC—prostate cancer, PI3K—phosphatidylinositol 3-kinase, PSA—prostate-specific antigen, RARα—retinoic acid receptor α, ROS—reactive oxygen species, SAHA—suberoylanilide hydroxamic acid, ST1926—adarotene, TFN—teriflunomide, TGI—tumor growth inhibition, TR4—testicular nuclear receptor 4, VEGF—vascular endothelial growth factor, cPARP—cleaved poly(ADP-ribose) polymerase, eIF4E—eukaryotic translation initiation factor 4E, fAR—full-length androgen receptor, mtDNA—mitochondrial deoxyribonucleic acid, n/c—no change, p—phosphorylated protein, ↑—an increase in given entity was observed, ↓—a decrease in given entity was observed.

**Table 2:** Summary of clinical studies that investigated the effectiveness of fenretinide in the treatment of PC (published between 2009 and 2021).

Number of PC Cases	Patients	Dose of Fenretinide	Duration of the Study	Study Endpoints	Results	Reference
23	Patients with PSA ≥2 ng/mL after radical prostatectomy and/or radical radiotherapy (with metastases excluded). Ethnicity: Americans Age: 69 years (median)	900 mg/m^2^ of body surface area twice daily for 1 week every 3 weeks	1 year, follow-up: 17.7 months (median)	PSA decline ≥50% or ≥5 ng/mL PSA-stable disease Time to PSA progression Probability of no PSA-progression in 6 months	0% 30% (95%CI: 14–52%) 4.6 months (95%CI: 3.2–8.2) 0.37 ± 0.10	Cheung et al., 2009 [60]
27	Patients after castration with rising PSA >10 ng/mL. Ethnicity: Australians, Asians Age: 74 years (median)	900 mg/m^2^ of body surface area twice daily for 1 week every 3 weeks	1 year	PSA decline >50% for at least 3 weeks PSA-stable disease for 6 weeks Time to treatment failure (PSA-based assessment)	4% (maximum of 39 days) 52% 54 days	Moore et al. 2010 [58]

Abbreviations: PC—prostate cancer, PSA—prostate specific antigen.

## Abbreviations

4E-BP1	4E-binding protein
4HPR	*N*-(4-hydroxyphenyl) retinamide
5-AzadCyD	5-aza-2′-deoxycytidine
AR	androgen receptor
ATRA	all-trans-retinoic acid
AnRE	antioxidant response element
Bcl-2	B-cell lymphoma
BRCA1/2	breast cancer 1/2
CyB	cyclin B
CyD1	cyclin D1
CI	combination index
CRPC	castration resistant prostate cancer
DHODH	dihydroorotate dehydrogenase
DNA	deoxyribonucleic acid
E-cad	E-cadherin
ERG	ETS-related gene
ETS	erythroblast transformation specific
GSK-3β	glycogen kinase synthase-3β
HDAC	histone deacetylase
HIF-1α	hypoxia inducible factor-1α
IC_50_	half-maximal inhibitory concentration
IGF-1	insulin-like growth factor-1
MMP	matrix metalloprotease
Mnk1/2	mitogen-activated protein kinase interacting kinases 1 and 2
N-cad	N-cadherin
Nrf2	nuclear factor-erythroid 2 p45-related factor 2
PARP	poly(ADP-ribose) polymerase
PC	prostate cancer
PI3K	phosphatidylinositol 3-kinase
PPAR	peroxisome proliferator-activated receptor
RAMBA	retinoid acid metabolism blocking agent
RAR	retinoid acid receptor
RARE	retinoid acid response element
RNS	reactive nitrogen species
ROS	reactive oxygen species
RXR	retinoid X receptor
SAHA	suberoylanilide hydroxamic acid
SIRT	sirtuin
SOD	superoxide dismutase
TR	thyroid receptor
TR4	testicular nuclear receptor 4
VDR	vitamin D receptor
VEGF	vascular endothelial growth factor
eIF4E	eukaryotic translation initiation factor 4E
mTOR	mammalian target of rapamycin
